# Profiling DNA damage in 3D Histology Samples

**DOI:** 10.1007/978-3-031-16961-8_9

**Published:** 2022-09-15

**Authors:** Kristofer E. delas Peñas, Ralf Haeusler, Sally Feng, Valentin Magidson, Mariia Dmitrieva, David Wink, Stephen Lockett, Robert Kinders, Jens Rittscher

**Affiliations:** 1 Department of Engineering Science, University of Oxford, United Kingdom; 2 Nuffield Department of Medicine, University of Oxford, UK; 3 Big Data Institute, University of Oxford, Li Ka Shing Centre for Health Information and Discovery, Oxford, UK; 4 Department of Computer Science, University of the Philippines, Philippines; 5 Frederick National Laboratory for Cancer Research, National Cancer Institute, USA; 6 Center for Cancer Research, National Cancer Institute, USA

**Keywords:** cell morphology, DNA damage, 3D histology, representation learning

## Abstract

The morphology of individual cells can reveal much about the underlying states and mechanisms in biology. In tumor environments, the interplay among different cell morphologies in local neighborhoods can further improve this characterization. In this paper, we present an approach based on representation learning to capture similarities and subtle differences in cells positive for *γ*H2AX, a common marker for DNA damage. We demonstrate that texture representations using GLCM and VAE-GAN enable profiling of cells in both singular and local neighborhood contexts. Additionally, we investigate a possible quantification of immune and DNA damage response interplay by enumerating CD8+ and *γ*H2AX+ on different scales. Using our profiling approach, regions in treated tissues can be differentiated from control tissue regions, demonstrating its potential in aiding quantitative measurements of DNA damage and repair in tumor contexts.

## 1 Introduction

The analysis of cell morphology is an important task in biology and is an active area of research where techniques are being developed for different imaging modalities and domains [6]. The characterization of morphology can give clues on cell functions and responses [9, 15]. Different cellular processes can alter the morphology of the cell. Importantly, morphology allows assignment of biological effects due to, for example, drug treatment, to be assigned to specific cell types in a tissue under study. This process is performed by pathologists and automated methods that can help them profile morphology of cells and tissues can have big impact in experimental medicine.

One cell process that manifests differently, in terms of morphology, due to many underlying factors is DNA damage and repair. Normally, a DNA-damaged cell will initiate repair once it detects alterations such as breaks, fragmentation, translocation and deletions in the DNA. If excessive damage has occurred, programmed cell death can be triggered. In tumor contexts, studying DNA repair mechanisms is specially important as evidences of impaired repairing capability in tumor cells have been presented [1]. Dysregulation of DNA damage response (DDR) promotes genomic instability, increased mutation rate, and intra-tumor heterogeneity [4, 3]. Inhibition of DNA repair by targeting specific repair enzymes, e.g. topoisomerases and PARP, has proven to be a useful strategy for treating patients with solid tumors, and a number of drugs are widely used in clinical practice.

Our understanding of DDR defects in tumors is not yet complete but several anti-cancer therapies already exploit this mutation. Ionizing radiation and chemotherapy aim to induce cell death by damaging DNA. This process however is not guaranteed as tumor cells can still initiate DNA repair pathways and become resistant to these therapies. Inhibiting such pathways and specific targeting of tumor cells can therefore enhance the anticancer effect of DNA damage-based therapy [17].

To further efforts in this area, the quantification of DNA damage is the subject of many previous studies. DNA damage in cells is frequently investigated through imaging with a marker for *γ*H2AX [13, 20, 7, 8, 24]. Traditionally, the quantification is done by counting the foci in cells either manually or automatically, or by counting the number of cells in an image field that are positive for *γ*H2AX signal above some cutoff value assigned on a per cell basis [2, 23].

In this work, we investigated the use of representation learning and texture features computed from fluorescence intensity statistics (gray-level co-occurrence matrices, GLCM) and latent encoding using a deep learning model (VAE-GAN) towards building profiles of cells in 3D histology. Representation learning reduces the need for manual counting and measurements to capture similarities and dissimilarities in data points. Using these discovered features from representation learning in an unsupervised manner enables building phenotypes without the need for annotation or additional fluorescent markers. We deviate from previous works by profiling DNA damage using visual texture, instead of directly computing for foci density. We analyzed 3D volumes of 125*μ*m thick 4T1 tissue sections, identified cells and cell clusters with DNA damage, and extended the characterization to local neighborhoods of cells, looking at proximity of immune cells (CD8+) and *γ*H2AX+ cells in both control and treated samples.

We highlight the contribution of this work as follows:

1. State-of-the-art artificial intelligence methods are integrated with thick 3D histology analysis by developing a seamless pipeline, from segmentation of 3D 4T1 histology volumes to profiling DNA damage in local neighborhoods of cells.
2. Representations of morphological variations of DNA damage in cells are constructed using a VAE-GAN model and GLCM features.
3. Clustering on the extracted features using GLCM and VAE-GAN provided pseudo-labels for texture classes that can be used to characterize tissue regions.
4. Local neighborhood profiles that were constructed using our analysis pipeline and the proximity information on CD8+ and *γ*H2AX+ cells reveal differences between control and treated classes, demonstrating potential in refining quantification of experiments.

## 2 Methods

### 2.1 Data

In our experiments, we analyzed four 4T1 mouse tumor tissue samples. Thick tissue biopsy sections (125*μm*) were cleared and imaged using standard confocal microscopy at 63*×* magnification. Tissues were treated with irradiation, indomethacin, and L-NAME. Cells were stained for *γ*H2AX, CD8, pan proteins, and DAPI.

From the acquired 3D confocal images, regions of size 302px×302px×130px (W×H×D) with high level of fluorescence for *γ*H2AX and CD8 were manually curated and extracted. Twelve sub-volumes were selected from the control and 14 sub-volumes were selected from the treated samples. The number of cells in each selected region ranged from 500 to 900.

### 2.2 Segmentation

**Fig. 1:**
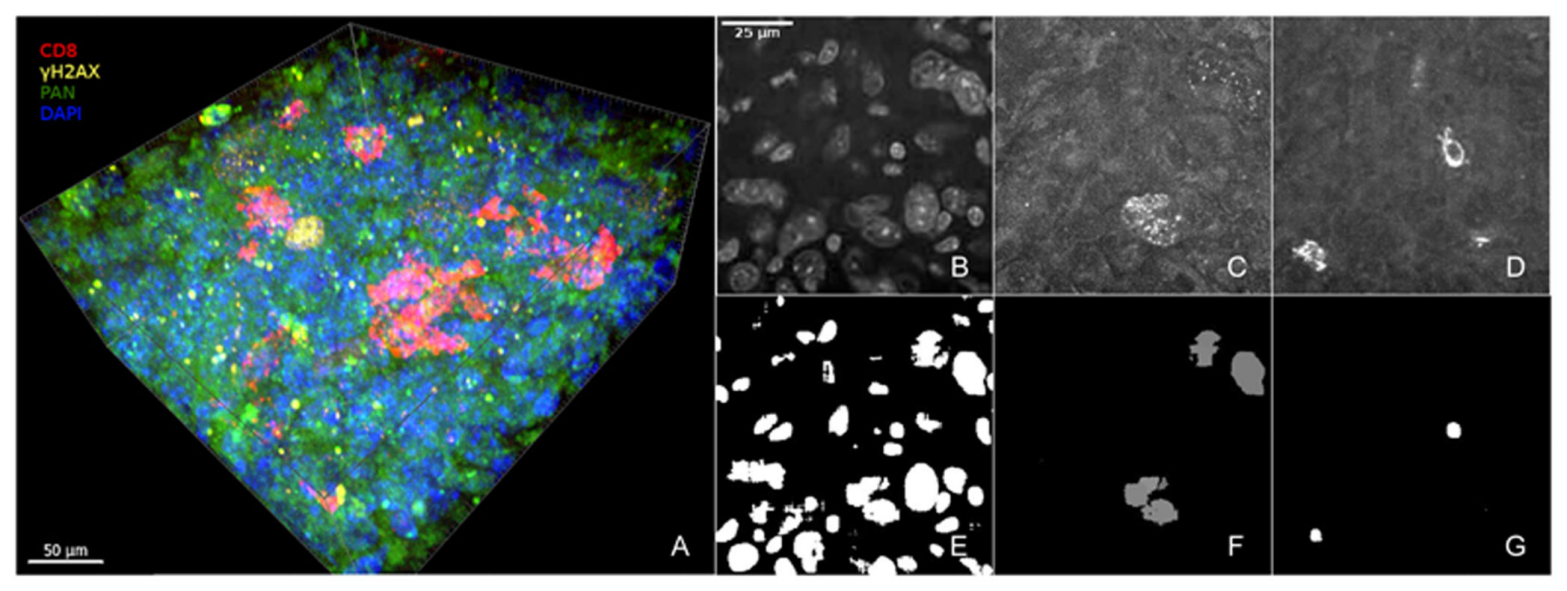
A 3D image volume (A) from the dataset used in this work (CD8 in red, *γ*H2AX in yellow, pan protein in green, DAPI in blue), *z*-slices of individual channels (B-D) and nuclei segmentation (E-G). Using the DAPI channel (B), Cellpose was used to generate segmentation of all nuclei (E). Using this segmentation and threshold masks on the *γ*H2AX channel (C) and the CD8 channel (D), *γ*H2AX+ cells and CD8+ are identified (F,G).

The first step in our analysis is to isolate individual nuclei in the 3D volumes, one of which is shown in Figure 1A. We used a pre-trained Cellpose [22] model for this segmentation task. Cellpose is a segmentation model for biological images based on deep learning. The segmentation is done by estimating flow gradients from cell centroids and reconstructing cell outlines by tracking these gradients. The model has been demonstrated to generalize well to many different microscopy data and can be used without the need for retraining. Figure 1E shows a slice of a 3D Cellpose nuclei segmentation of a histology volume using its DAPI channel (shown in Figure 1B).

After segmenting the nuclei using Cellpose, we wanted to focus on cells positive for *γ*H2AX (see Figure 1C). We observed that simple thresholding on the *γ*H2AX channel is sufficient to segment *γ*H2AX+ nuclei and using the Cellpose model on the *γ*H2AX channel actually resulted in “hallucinated” nuclei, false segmentations in areas without nuclei. One drawback of the thresholding, however, is that cell outlines are rougher compared to the Cellpose output. To solve this, we superimposed the thresholded *γ*H2AX volumes with the previous nuclei segmentation. Cells with a significant overlap in this superimposition are retained in the final *γ*H2AX segmentation mask. Figure 1F shows segmented *γ*H2AX+ nuclei.

For each of the identified *γ*H2AX+ nuclei, we extracted bounding boxes to be used for the computation of texture feature representation. A total of 372 *γ*H2AX+ nuclei were extracted with high confidence. Augmentation was done to increase this number for training our model.

We also applied a similar segmentation approach to the CD8 channel (see Figures 1D and 1G) to isolate CD8+ cells. As the immunomarker for CD8 binds on the cell membrane, the nuclei segmentation was dilated first before superimposition to ensure overlap.

### 2.3 Building cell profiles of DNA damage

Techniques that look solely at shape like in [18] exist and were demonstrated to capture the heterogeneity and variations of cells in tissues. However, they rely heavily on segmentation. In our case, as *γ*H2AX+ is primarily analyzed for its distribution across nuclei, texture more than shape would capture this information.

#### Statistical Texture Features

A popular way to quantify texture in images is to compute and analyze gray-level co-occurrence matrices (GLCM). GLCM is a second-order statistic that captures pairwise relationship of intensity levels within a specific neighborhood size in an image. Its use in medical image analysis is widespread, ranging from brain magnetic resonance images [21] to liver ultra-sound [25]. From the computed matrices, features encapsulating the intensity co-occurrences in different ways can be measured [12]. In this work, we used the following GLCM features: energy, contrast, prominence, and correlation.

#### Latent Features

We also explored the use of latent features from deep learning methods to characterize the morphology of DNA-damaged and apoptotic cells. Data representations in latent spaces have been applied to many biological domains and were demonstrated to capture subtle similarities and dissimilarities in imaging data [19, 5]. In this work, we used a type of a variational autoencoder [14].

**Fig. 2:**
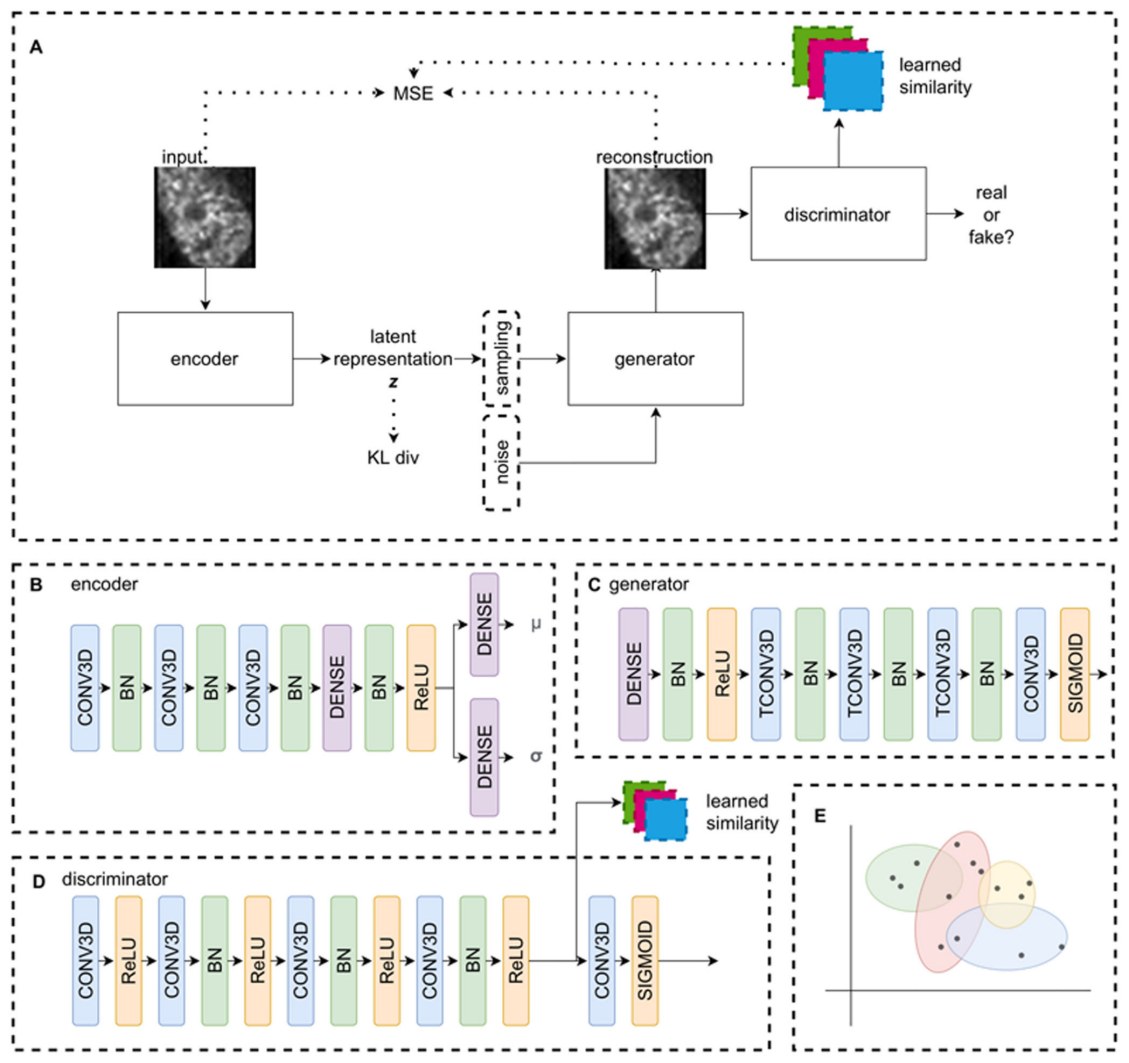
The VAE-GAN architecture (A) used to construct the manifold of DNA damage in cells. The network is composed of three components: encoder, generator, and discriminator. The encoder (B) maps input images to 16-dimensional Gaussian distributions with diagonal covariance. The generator (C) produces a reconstruction from sampled points in the latent space. The discriminator (D) forces the generator to output images as similar to the input as possible. Latent encoding of the image volumes are then clustered to form pseudo-classes of DNA damage subtypes (E).

VAE-GAN [16] is a type of a variational autoencoder that adds a discriminator to the network to further improve decoding and stabilize the training process. It borrows the discriminator concept from generative adversarial networks (GAN) [10], another generative deep learning technique. Further, it utilizes a learned similarity metric derived from feature maps from the discriminator in addition to the pixel-wise MSE loss. As capturing good representations of DNA damage-induced morphology and not faithful reconstructions of image data is the primary task, a high level similarity test is desirable. For this reason, we employed the VAE-GAN architecture to construct a manifold for DNA damage. Figure 2 shows the structure of our model. Our model’s encoder contains three convolutional layers with batch normalization, followed by a series of dense feed-forward layers. For all our experiments, the dimension of the latent space is set to 16. The generator/decoder mirrors the encoder with three transposed convolutional layers. For the discriminator, a network with five convolutional layers with batch normalization was constructed. Feature maps after the fourth convolutional layer are extracted and used for the learned similarity metric. For training, the images of segmented *γ*H2AX nuclei were resized to and centered on 64 × 64 patches, and scaled to the [0,1] range. All network modules are trained with ADAM optimizers with learning rate of 0.0001 for 500 epochs.

### 2.4 Pseudo-class labels

Extracted representations for individual cells were clustered into 5 pseudo texture classes using KMeans. These clusters are then projected and visualized into 2D using principal components analysis. Since our data lacks expert annotation for repair and apoptotic classes, we aimed to use the formed clusters to find texture similarities in foci across individual cells, acting as class label surrogates.

### 2.5 Comparing cell profiles in control and treated tissues

The last step in our analysis is to form profiles of local neighborhoods of cells to distinguish between regions from treated and control samples. Constructing spherical neighborhoods of various radii lengths, centered on identified *γ*H2AX+ cells, we looked at the proximity of other *γ*H2AX+ from different texture classes. We also counted the number of identified CD8+ cells within the vicinity. This colocalization of *γ*H2AX+ and CD8+ cells follows the results of studies establishing links between immune response and DNA repair in tumor microenvironments [11].

## 3 Results and Discussion

**Fig. 3:**
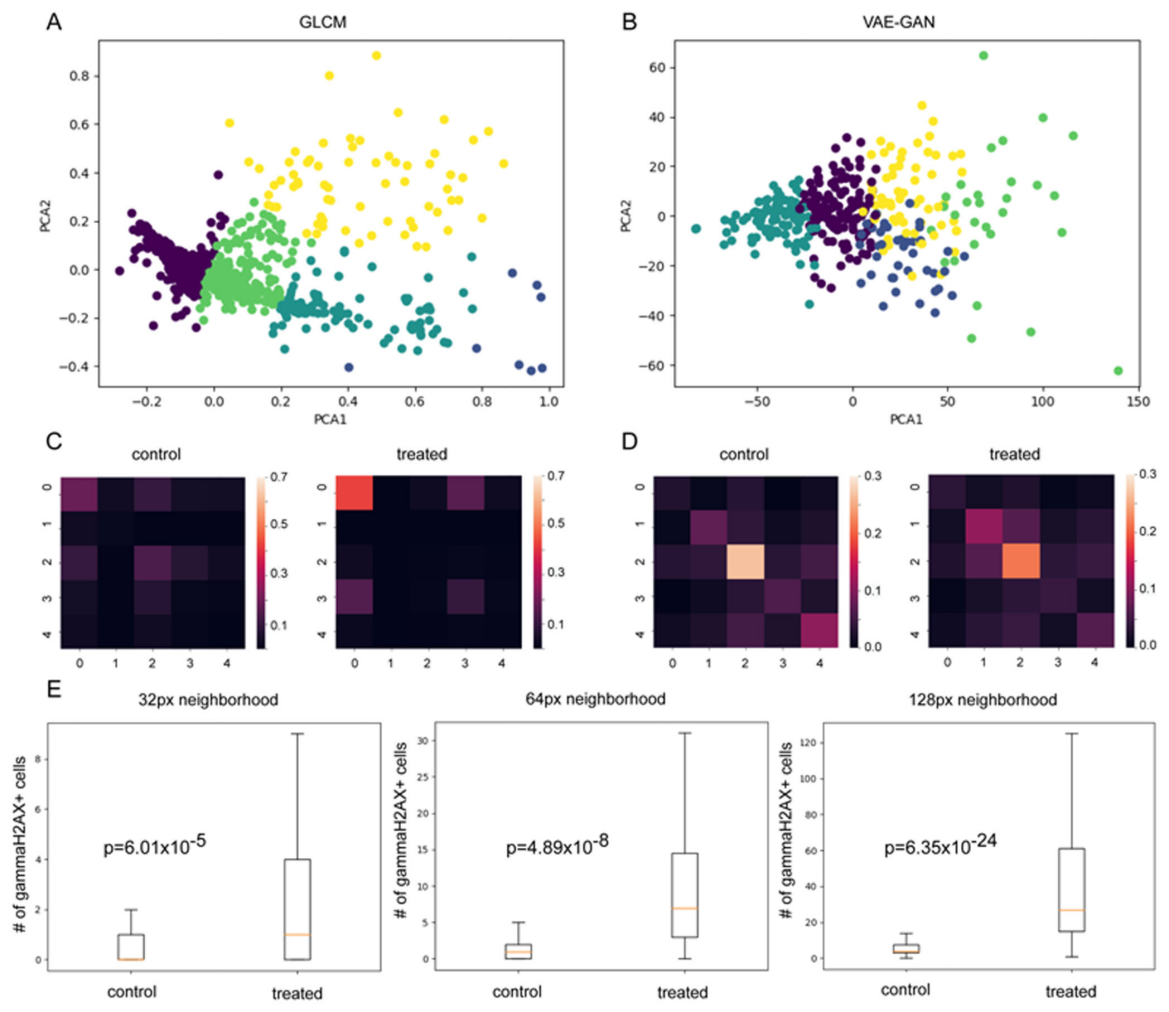
Clustering on GLCM representations (A) and VAE-GAN encodings (B). Heatmaps, using pseudo-classes from GLCM (C) and VAE-GAN(D), show differences in *γ*H2AX profiles in neighborhoods in control and treated samples. Proximity analysis shows a higher number of *γ*H2AX+ cells near CD8+ cells in treated samples (E).

Using the texture subtypes identified by clustering on GLCM-based features, we constructed heatmaps for control and treated samples. Shown in Figure 3 are co-occurrence heatmaps of texture subtypes. Visually, there seems to be a clear difference between control and treated tissues. Clusters of *γ*H2AX+ cells with labels 0 and 3 are more prominent in treated tissues. On the other hand, control tissues exhibit clusters of *γ*H2AX+ cells with labels 0 and 2. We also observed difference in heatmaps using pseudo-class labels generated from clustering VAE-GAN encodings as shown in Figure 3. The number of neighboring *γ*H2AX+ cells with labels 2 and 4 are elevated in control tissues while prominence in treated is in *γ*H2AX+ cells with labels 1 and 2. Both representations resulted in distinct profiles for control and treated regions. This demonstrates that the identified texture subtypes capture information that can be utilized for a more refined analysis of tissue sections. Lacking annotation for the data used in this work, however, explaining the profile differences will be difficult. It will be worth exploring how these texture subtypes correspond to DNA damage response types.

Lastly, we looked at the proximity of identified CD8+ cells to *γ*H2AX cells. Our understanding of the links between immune response and DNA repair still contain gaps and tools that can provide quantitative measurements have the potential to accelerate current studies in this area. In Figure 3, we enumerated *γ*H2AX+ cells within the neighborhood of CD8+ cells. We computed this on various scales (32px (10.28*μ*m), 64px (20.56*μ*m), 128px (41.13*μ*m)). In general, we see an increase in the number of *γ*H2AX+ cells near CD8+ cells. While further tests are needed for increased immune response to be claimed with certainty, quantifying the proximity of these two cell types can be useful in establishing baseline levels and investigating the heterogeneity in tumor tissues.

## 4 Conclusion

Here we presented a pipeline for the analysis and profiling of 3D histology samples. We demonstrated using a 4T1 dataset that texture representations using GLCM and VAE-GAN have a potential impact on DNA repair analysis on tissue microenvironments. Using our approach, we identified *γ*H2AX+ and CD8+ nuclei and constructed profiles describing local cell neighborhoods of different scales. The learned texture subtypes and the neighborhood profiles show no-table differences between control and treated tissues. This can enable a more precise quantification of experiments, particularly response to anti-cancer drugs and therapies.

In future work, we aim to validate the texture clusters formed from GLCM and VAE-GAN features against morphological manifestation of DNA repair and apoptosis types. While the pseudo-class labels from these clusters are demonstrated to be useful in showing differences between regions in treated and control tissues, it is desirable to improve the explainability of the labels and ground them on current biological and pathological knowledge. This, however, would entail curating individual cells and incorporating additional markers to determine the specific types. Moreover, we envision to extend our profiling to other specific cell targets such as immune cells and to support further studies looking at links between immune response and DNA repair in tumor contexts.
